# Study on Height Measurement for Polyethylene Terephthalate (PET) Materials Based on Residual Networks

**DOI:** 10.3390/s25134030

**Published:** 2025-06-28

**Authors:** Chongwei Liao, Weixin Zhang, Yujie Peng, Changjun Liu

**Affiliations:** 1 School of Electronics and Information Engineering, Sichuan University, Chengdu 610064, China; 2 Yibin Industrial Technology Research Institute of Sichuan University, Yibin 644000, China

**Keywords:** microwave drying, PET, power measurement, MLP, residual networks, equivalent permittivity, vector network analyzer

## Abstract

In industrial production, high-power microwaves are commonly used for heating and drying processes; however, their application in measurement is relatively limited. This paper presents a power measurement system to enhance the use of microwave measurements in industry and improve the efficiency of microwave drying for PET particles. Operating at 2.45 GHz, the system integrates four-port power measurements based on the multilayer perceptron (MLP). By introducing residual connectivity, the residual network is determined to detect the height of PET particles. Experimental results show that this system can perform rapid measurements without needing a vector network analyzer (VNA), significantly improving the efficiency of microwave energy utilization in the early drying stages. Furthermore, the system offers practical and cost-efficient predictions for low-loss particulate materials. This power measurement strategy holds promising application potential in future industrial production.

## 1. Introduction

PET is a synthetic material commonly used in packaging and cosmetics due to its excellent mechanical properties, water resistance, and recyclability [1]. In 2020, the annual production of PET reached approximately 300 million tons. Over 90% of plastics were derived from petroleum-based materials, contributing to 20% of total petroleum consumption [2]. In 2019, global plastic waste generation was estimated at 353 million metric tons, and projections suggest it could triple by 2060. As a result, the demand for PET is increasing every year, leading to significant energy consumption [3].

Various industry initiatives and private companies have committed to increasing their products’ recycling rate and recycled content, aiming for 30% by 2025 and 65% of PET packaging materials to be recycled by 2030 [4]. The rapid heating properties of microwaves make them suitable for applying in producing and recycling plastics [5]. Qiang Tang and Jau Tang et al. studied the combustion process of butane in a microwave plasma torch, which provides a reliable solution for exhaust gas treatment and plastic recycling [6]. Before extrusion for film packaging, masterbatch or recycled pellets of PET must be dried to remove moisture, which consumes a great deal of time and energy [7]. The strong interaction between microwaves at 2.45 GHz and water molecules can produce higher temperatures, which can enhance water escape efficiency on one hand and enable higher energy level densities on the other [8]. Recycling PET occurs in a specific reactor, which utilizes microwave radiation to depolymerize the initial polymer rapidly. At the end of the depolymerization process, microwave drying is performed in an Agitated Nutsche Filter (ANF). Leveraging realistic measurements of mixture composition and mathematical modeling of dielectric material properties based on concentration and temperature, microwave drying time is reduced instead of expensive dynamic impedance matching adjustments [9].

PET can be used as a material with low permittivity and dielectric loss as a substrate material for microwave-integrated circuits or communication-related electronic components [10]. However, the lower permittivity results in better reflections and lower efficiency for microwave drying of PET materials [11]. In particular, proper drying parameters before extrusion and the sensitivity of the material to moisture can lead to degradation [12]. Microwave drying technology is generally used with high moisture content in food processing. Heri Septya Kusuma et al. studied the microwave drying characteristics of Moringa oleifera leaves at 136 W and 264 W [13]. Nan-nan An et al. created an overview of future research directions in microwave drying for food applications in terms of artificial intelligence, renewable energy, and computational fluid dynamics technologies [14].

In recent years, relatively little research has been done on microwave drying for PET materials. Since moisture adversely affects PET’s characteristic viscosity, producing high-quality preforms requires very high viscosity. In 1995, C. A. R. Anjos et al. studied the contribution of microwave heating to drying speed, quality control, and overall capitalized cost [15]. The drying kinetics of PET were investigated, but the measured data matched the calculated data slightly poorly [16]. Later, using an industrial-scale prototype machine of the microwave drying system, five different polymers were tested to obtain the interdependence between polymer properties (e.g., thermal properties and molecular structure) as well as the drying kinetics of the respective polymers [17]. To investigate the possibility of using a frequency-controlled microwave source (i.e., solid-state microwave generator) for fast and efficient heating of PET, numerical simulations of microwave heating of PET were performed by Paolo Veronesi et al. based on the permittivity of PET [18]. A comparison of the energy required to reduce the moisture content of PET flakes in an electric oven and a microwave has been researched, illustrating that microwave reduces drying time and energy consumption by up to 18% [19].

Due to the inherent limitations of materials, permittivity measurements are utilized in medical microwave imaging, temperature detection, electromagnetic imaging, and the prediction of soil properties. [20,21,22,23,24]. The material permittivity equation can be extracted from the scattering data using curve-fitting techniques based on the measured parameters [25]. PET, a low-loss dielectric material, shows low microwave sensitivity, resulting in significant reflection power. Therefore, it is crucial to monitor both the power and the volume, as this information aids in predicting the material’s state. In this paper, we designed a microwave drying system precisely to monitor the reflection power. By integrating this system with the MLP with residual network technology, we can predict the height of the PET material for effective drying control. For the first time, we have realized the network mapping relationship between the reflected power of the cavity and the state of the material with four feed ports, which provides an efficient, low-cost, and highly integrated real-time power monitoring solution for the future microwave drying of low moisture content materials.

## 2. Methods

### 2.1. Reflection Power Measurement System

**Figure 1:**
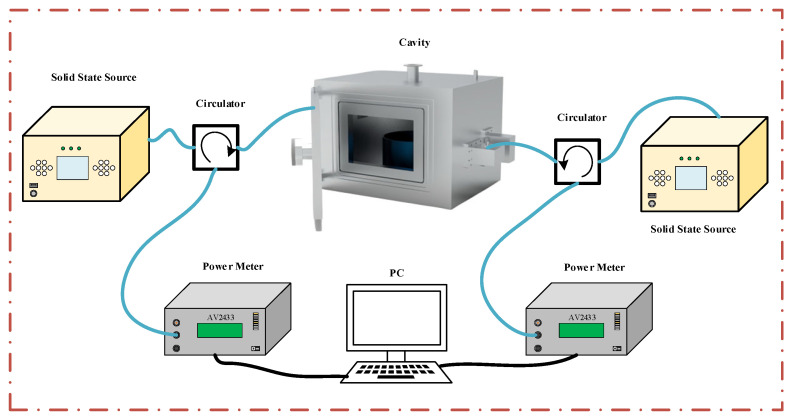
Reflected microwave power measurement system.

We conducted reflection power measurements on PET materials using a four-port cavity at various heights. As shown in Figure 1, the schematic diagram of the measurement system consists of a metal shielding cavity, four feeding ports connected by waveguide coaxial converters, a circulator for connections, a solid-state source, and a power meter. The system operates at a frequency of 2.45 GHz with a power output of 10 W. The dimensions of the metal shielding cavity are 0.6 m × 0.44 m × 0.44 m. The amplitude of the reflection power was measured with a power meter (AV2433, Ceyear, China) without a vector network analyzer. We tested the four-port reflection power at heights ranging from 0.01 m to 0.16 m, using a diameter of 0.257 m.

**Figure 2:**
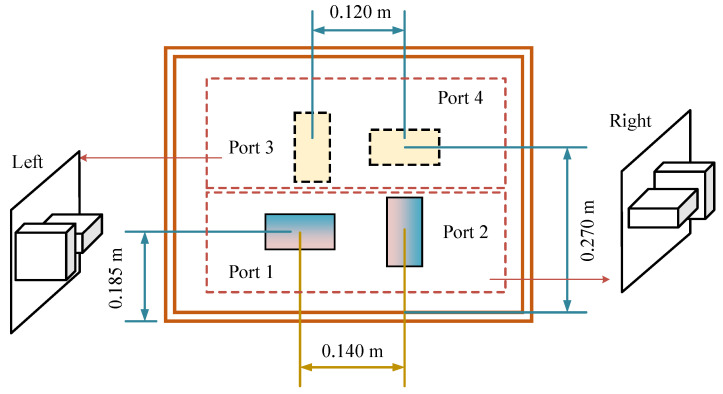
Positions of the four feeding ports.

A two-by-two, vertically distributed structure was designed to minimize the coupling between the ports, as illustrated in Figure 2. Ports 1 and 2 are positioned perpendicularly on one side and perpendicular to ports 3 and 4. The distance between the centers of ports 1 and 2 is 0.14 m, which is greater than the 0.12 m between ports 3 and 4.

### 2.2. Microwave Simulation

As a low-loss dielectric material, the volume and permittivity of PET particles are crucial parameters in microwave processing. At room temperature, their ability to absorb microwaves is weak. However, as the temperature increases, the real and imaginary permittivity components rise [26]. Due to the presence of air between particles, the acquisition of the equivalent permittivity facilitates the study of the material state. According to the Li equation [27], the equivalent permittivity of PET particles and air is calculated as follows:

(1) $$\ln\varepsilon_{eff}=f\ln\varepsilon_{1}+(1-f)\ln\varepsilon_{2}$$

where $\varepsilon_{eff}$ is the equivalent permittivity, $\varepsilon_{1}$ is the permittivity of PET materials, and $\varepsilon_{2}$ is the permittivity of air. By comparing the mass and density, the volume fraction of PET and air is denoted as *f* and 1 − *f*, respectively.

Unlike other equivalent dielectric models, this model treats the real and imaginary parts of the dielectric response logarithmically, incorporating weight factors based on the volume ratios of particulate matter and air gaps. The resulting real and imaginary components of the dielectric constant are lower than those calculated by the Complex Refractive Index (CRI) model and the Landau–Lifshitz–Looyenga (LLL) model. Additionally, the dimensions of the particle model directly influence the reflected energy at the feeding port. To address this, the Li equation is employed to mitigate the effect of the particle modelling size. By reducing energy absorption by the particles, the particle size can be appropriately increased, which achieves a balance between computational efficiency and accuracy.

**Figure 3:**
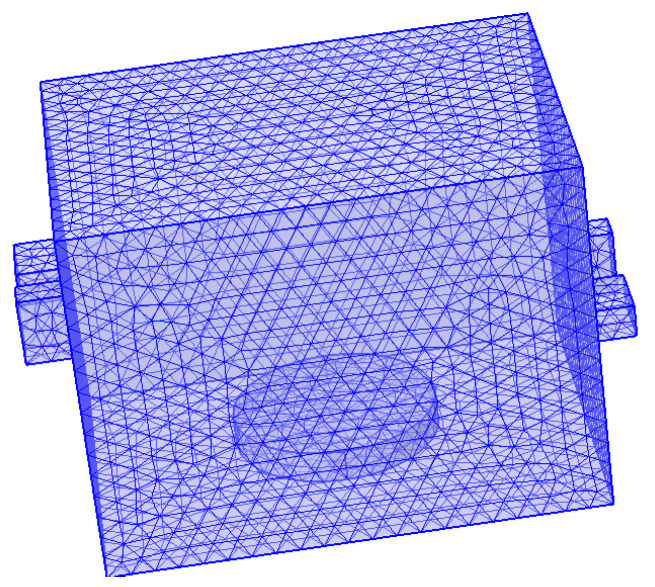
3D model of the shielded cubic cavity.

After conducting calculations, the real and imaginary parts of the cylinder made of PET particles are 1.96 and 0.014, respectively. This indicates that the cylinders do not absorb microwaves. Consequently, a cubic cube is modeled for finite element analysis, as illustrated in Figure 3. The whole model was subjected to finite element analysis (FEA) in COMSOL Multiphysics^®^ software (version 6.3). Set the PET material model as a solid heat transfer module and set the boundary as thermally insulated. Considering that the particles are cylindrical, we set ideal air to fill the gaps between particles. The material of the rectangular cavity is set to aluminum, and the resonant cavity boundary condition is set to an impedance boundary condition to improve the simulation efficiency. Since a PET particulate matter is much smaller than the operating wavelength, we perform free tetrahedral meshing in different dimensions for the cavity and the load, respectively. The largest cell size is 0.0747 m, the smallest cell size is 0.0135 m, the maximum cell growth rate is 1.5, and the narrow region has a rate of 0.5, respectively. The finite element simulations of all models were performed on a workstation configured with an Intel (R) CPU i9-13900K and 128 GB of RAM.

**Figure 4:**
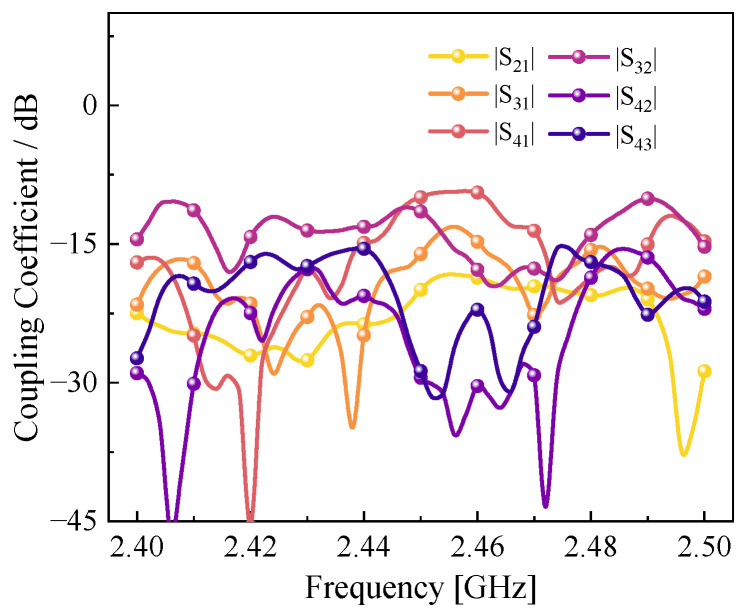
The coupling coefficient between four ports in the 2.4 GHz to 2.5 GHz range.

To accurately reflect the materials’ boundary conditions, simulations were conducted on the coupling between different ports, as shown in Figure 4. The inter-port coupling is limited to around −15 dB, which ensures that the reflected power of each port carries the materials’ scattering information at different height levels.

**Figure 5:**
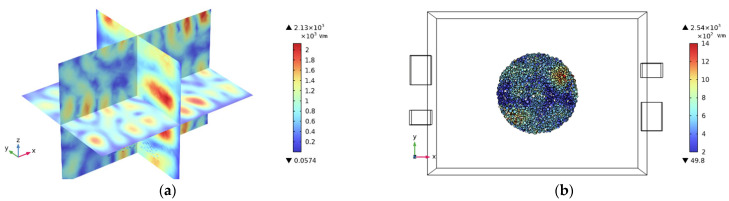
(**a**) The electric field distribution inside the cavity is unloaded. (**b**) The electric field distribution of PETs with a height of 1 cm.

**Figure 6:**
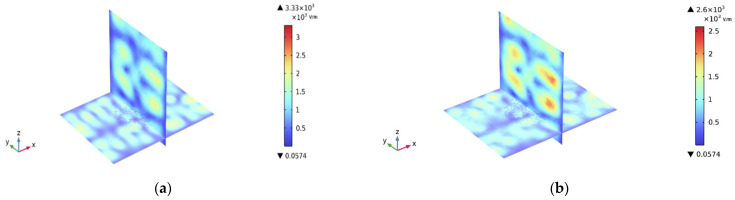
Electric field distribution inside the cavity distributions loaded with a height of (**a**) 1 cm of PETs and (**b**) 3 cm of PETs.

By modeling the particles, we simulate different heights of stacked materials to obtain large amounts of reflected power for training. As Figure 5 and Figure 6 show, when the load height is varied, the electric field distribution in the cavity changes. The optimal load shape can be determined based on the received reflected power of the four ports at different load heights. $TE_{mnp}$ and $TM_{mnp}$ are the electromagnetic field modes in a rectangular resonant cavity. Assuming that the length, width, and height are *a*, *b*, *l*, respectively. The electromagnetic field densities of $TE_{mnp}$ and $TM_{mnp}$ can be written as below:

(2) $$TE_{mnp}\left\{\begin{array}{l} H_{x}=-\frac{1}{k_{c}^{2}}\beta \left(\frac{m\pi }{a}\right)H_{mnp}\sin\left(\frac{m\pi }{a}x\right)\cos\left(\frac{n\pi }{b}y\right)\cos(\beta z)\\ H_{y}=-\frac{1}{k_{c}^{2}}\beta \left(\frac{n\pi }{b}\right)H_{mnp}\cos\left(\frac{m\pi }{a}x\right)\sin\left(\frac{n\pi }{b}y\right)\cos(\beta z)\\ H_{z}=H_{mnp}\cos\left(\frac{m\pi }{a}x\right)\cos\left(\frac{n\pi }{b}y\right)\sin(\beta z)\\ E_{x}=\frac{j\omega \mu }{k_{c}^{2}}\left(\frac{n\pi }{b}\right)H_{mnp}\cos\left(\frac{m\pi }{a}x\right)\sin\left(\frac{n\pi }{b}y\right)\sin(\beta z)\\ E_{y}=-\frac{j\omega \mu }{k_{c}^{2}}\left(\frac{m\pi }{a}\right)H_{mnp}\sin\left(\frac{m\pi }{a}x\right)\cos\left(\frac{n\pi }{b}y\right)\sin(\beta z)\\ E_{z}=0 \end{array}\right.$$

(3) $$TM_{mnp}\left\{\begin{array}{l} E_{x}=-\frac{1}{k_{c}^{2}}\beta \left(\frac{m\pi }{a}\right)E_{mnp}\cos\left(\frac{m\pi }{a}x\right)\sin\left(\frac{n\pi }{b}y\right)\sin(\beta z)\\ E_{y}=-\frac{1}{k_{c}^{2}}\beta \left(\frac{n\pi }{b}\right)E_{mnp}\sin\left(\frac{m\pi }{a}x\right)\cos\left(\frac{n\pi }{b}y\right)\sin(\beta z)\\ E_{z}=E_{mnp}\sin\left(\frac{m\pi }{a}x\right)\sin\left(\frac{n\pi }{b}y\right)\cos(\beta z)\\ H_{x}=\frac{j\omega \varepsilon }{k_{c}^{2}}\left(\frac{n\pi }{b}\right)E_{mnp}\sin\left(\frac{m\pi }{a}x\right)\cos\left(\frac{n\pi }{b}y\right)\cos(\beta z)\\ H_{y}=-\frac{j\omega \varepsilon }{k_{c}^{2}}\left(\frac{m\pi }{a}\right)E_{mnp}\cos\left(\frac{m\pi }{a}x\right)\sin\left(\frac{n\pi }{b}y\right)\cos(\beta z)\\ H_{z}=0 \end{array}\right.$$

where *m*, *n*, *p* are the number of complete half-cycle waveforms in the coordinate direction of *x*, *y*, *z*, respectively. Correspondingly, $E_{x}$, $E_{y}$, and $E_{z}$ are the scalar quantity components of the electric field in all directions. $H_{x}$, $H_{y}$, and $H_{z}$ are the scalar quantity components of the magnetic field in all directions. Propagation constants β=pπ/l characterize the propagation of electromagnetic fields in cavities.

### 2.3. Network Theory

An MLP, or Multilayer Perceptron, is a type of classical feedforward neural network and is one of the fundamental models in deep learning. It consists of several layers of neurons, including an input layer, one or more hidden layers, and an output layer. The main idea behind MLP is to learn complex function mappings through multiple nonlinear transformations across the layers, enabling it to tackle tasks such as classification and regression [28]. The MLP algorithm is widely used for post-error compensation in gravity gradient measurements, image recognition, and human activities or facial expression processing. It is also applied in indoor positioning systems, predicting porosity and clay volume fraction in geological logs, and forecasting photovoltaic power generation [29,30,31,32,33,34].

A residual network is a neural network architecture that utilizes a convolutional neural network to learn residual functions for more vigorous feature representation. Multiple residual blocks are stacked to form this network. Usually, each residual block is composed of multiple convolutional layers, a batch normalization layer, and an activation function [35].

**Figure 7:**
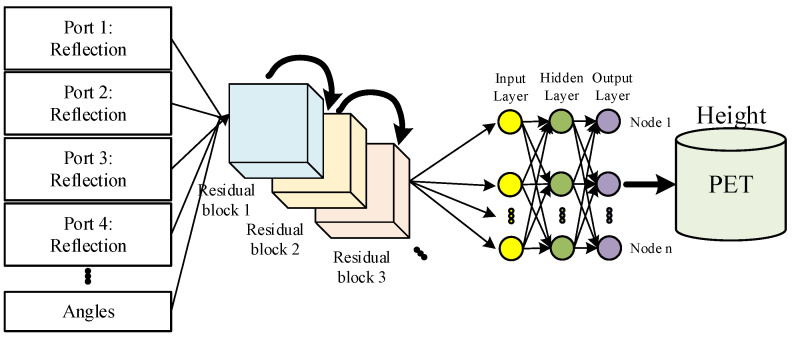
Structural diagram of the residual network based on the MLP.

In this paper, we designed a residual network combined with an MLP to reconstruct the state of PET particulates, as illustrated in Figure 7. Instead of using a Vector Network Analyzer (VNA), the measured reflection power is the network’s input feature. The input vectors in the input layer correspond to the reflection power from each port, while the output layer provides the height.

To improve the regression prediction ability of the network, the residual network was designed, which consists mainly of three residual blocks and a fully connected layer. The gradient vanishing problem is mitigated by introducing residual connectivity, allowing the network to train deep networks more efficiently. The activation function uses an exponential linear unit ELU, which enables the network to acquire the mapping ability of complex functions. The residual join is achieved by summing the output of the shortcut join with the production after a two-layer full join and batch normalization process. After the three residual blocks are stacked sequentially, the parameter information, such as reflected power, is fed into the MLP network for height prediction.

## 3. Results

### 3.1. Reflection Power Measurement

**Figure 8:**
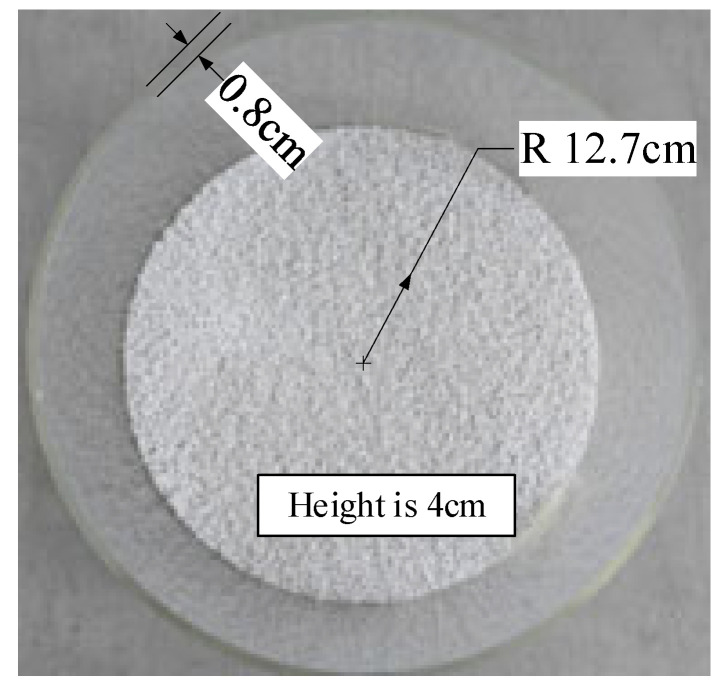
The sample diagram.

The selected granular masterbatch is an opaque white granular solid, and the size of the individual granules can be approximated as a cylinder with a height of 3 mm and a diameter of 1 mm, as shown in Figure 8. The quartz cavity was loaded with granules of different heights and flattened. The maximum height of the container is 17 cm, and the inner diameter is 25.4 cm. To represent volume changes, the height varied from 1 cm to 16 cm in 1 cm steps. The reflected powers of the four feeding ports showed different combinations when the cavity was loaded with different volumes of material.

**Figure 9:**
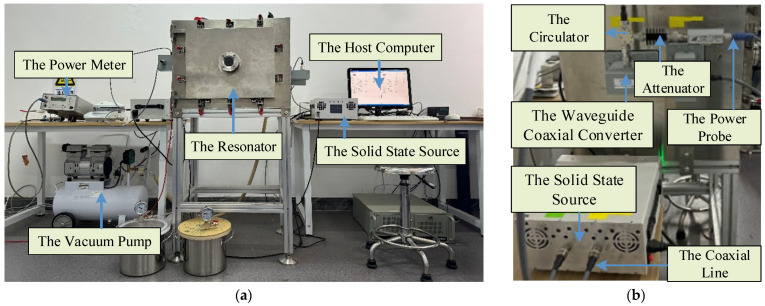
(**a**) The microwave power measurement system and (**b**) the port reflection monitoring module.

A four-port reflection measurement device has been developed, consisting of a microwave feeding module, a reflection power measurement module, and a network processing module. Figure 9a illustrates the entire system. The connector module, which includes the solid-state source and the power meter, is shown in Figure 9b and is connected via a circulator. The host computer transmits a control signal to drive a solid-state source to perform the frequency and power shifts. The microwaves were input to the circulator through the coaxial line. At this time, the forward-propagating microwave enters the resonant cavity, and the reflected microwave output from the cavity is reversed and input to port 3 of the circulator. The reflection microwave was input to the power meter through a 30 dB attenuator, which finally obtained the corresponding reflected power.

**Figure 10:**
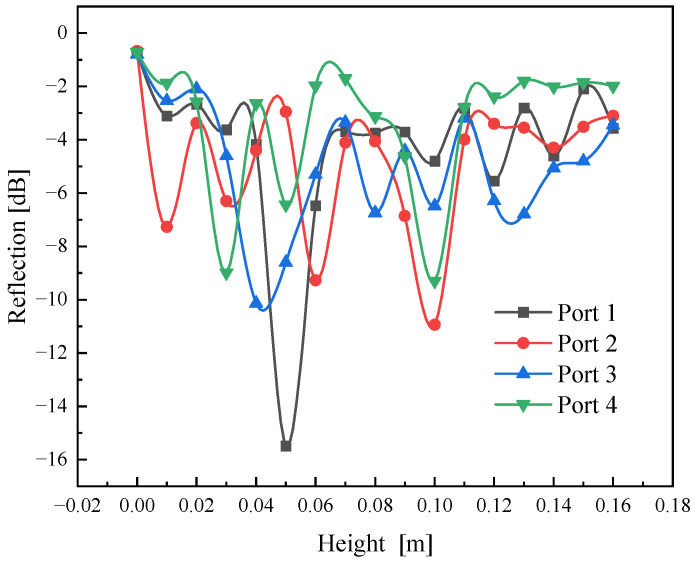
Variations of port reflections with height.

We performed four-port power measurements on materials positioned at different heights, and the variations in power are illustrated in Figure 10. Because the materials have low equivalent permittivity at room temperature, the reflection power from each port offers real-time insights into microwave scattering across these various heights.

PET particles’ equivalent permittivity and height correlate at a fixed working frequency of 2.45 GHz and room temperature. Using the MLP algorithm, we established a relationship between reflection power and volume. Based on the fitting curve for the equivalent permittivity of PET particles, we can predict the properties of PET materials using a power meter.

Although a VNA can fully capture the amplitude and phase information of reflected signals and indirectly reflect changes in cavity load volume, its high cost makes it unsuitable for real-time monitoring in industrial production processes. Meanwhile, integrating additional signal-receiving modules increases the overall system size. In contrast, a feedthrough-based real-time reflection monitoring strategy, requiring only a circulator and an external power meter, significantly improves production line integration and enables efficient utilization of feedthrough reflection data for rapid real-time monitoring.

When electromagnetic wave scattering, transmission, reflection, and heat conversion coincide, the power reflection signal carries nonlinear information that conventional analysis methods struggle to process to extract the required height-change characteristics. We established a correlation between material volume and feeder reflection power using a residual network to address this. By incorporating four-feeder spatial configurations and angular parameters, which consist of the line connecting the center points of the load and the feeder and the horizontal line, the prediction accuracy of the network model is enhanced.

### 3.2. Network Analysis

We propose a microwave–machine learning integration method based on residual networks, the core of which consists of three cascading residual blocks with a final classification layer. The architecture employs a cross-layer connection mechanism to alleviate the gradient vanishing problem and achieves efficient feature delivery through a dimensionality compression strategy. Each residual module follows a bilinear transformed architecture. The input features are first passed through a fully connected layer (FC1) with an exponential linear unit (ELU) activation function, which extends to 2048 dimensions, and then a quadratic linear transform is processed by the second linear layer (FC2). By gradually reducing feature dimensions, the outputs are quadratically batch-normalized and fused with the original inputs for information distillation. A learnable projection matrix achieves dimension alignment when the input and output dimensions are mismatched.

The main body of the network consists of a cascade of three decreasing residual modules: the first module maps the input features to a 2048-dimensional space, the secondary module performs dimensional compression to 1024 dimensions, and the final module further reduces to 512 dimensions. Each module successfully combines nonlinear transformations and cross-layer connections to form a feature reuse mechanism. The final mapping to the target dimension is completed through the fully connected layer (FC4). The output layer dimension was determined by four reflection power combinations, angles, and height. Experiments show that the structure achieves an effective balance between parameter efficiency and representation capability.

Based on the boundary conditions at different heights, the reflected power of the particles, and the incidence angles of the four ports, we obtained more than 3000 sets of data in finite element analysis, which were input into the residual network for training. The mapping relationship between the reflected power at different volumes was obtained. The corresponding incidence angles replace phase information, which shows good consistency between the predicted and actual values.

When comparing actual parameters to predicted parameters, it becomes evident that some prediction error is present. This error can result from several factors, including variations in scattering angles caused by the uneven surfaces of the particles and fluctuations in the output power of the solid-state source, both of which can impact measurement accuracy. Additionally, the precision of the measurement results can be affected by other factors, such as the height of the PET particles, variations in experimental temperature, and errors from the power meter and cables used in the measurements.

**Figure 11:**
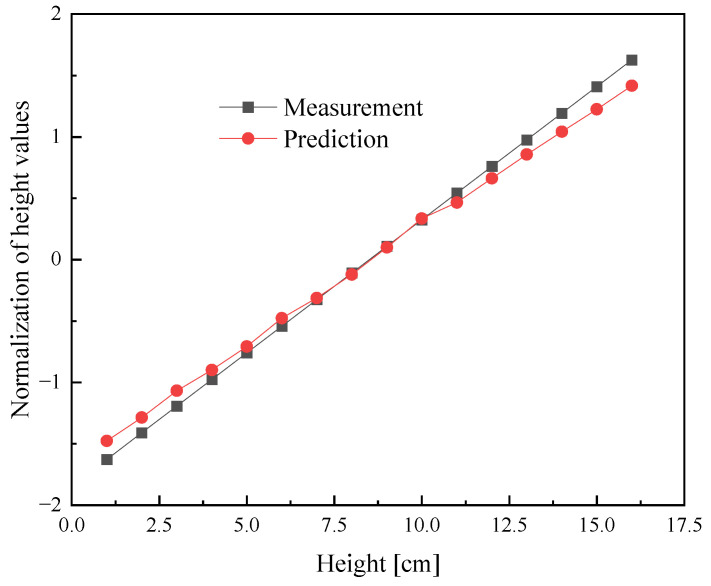
Curve fitting of the PET height based on the residual network.

As shown in Figure 11, there is a positive correlation between the predicted height and the measured value. This correlation demonstrates the accuracy and reliability of the residual network and the power measurement system, which were validated through experiments. Furthermore, using a quartz tube as the container for the PET particles, we successfully eliminated the impact of other materials on the amplitude of the reflected power based on the residual network. This approach effectively allowed us to measure the equivalent permittivity of other low-dielectric-loss particulate materials to inverse the height based on reflection powers.

## 4. Conclusions

This paper presents a PET material reconstruction system with a power measurement approach and a residual block reinforcement network based on an MLP. This system has been experimentally validated for its speed and accuracy in measuring particle states. Reflection power measurements were carried out for mixed particles at varying heights at four ports. The comparison between the experimental data and the predicted results demonstrated good consistency. The Mean Absolute Percentage Error (MAPE) of the model is about 1.81%, and the R-squared coefficient of determination reaches 0.99, which validates the predictive capacity of the network. The minor discrepancies observed may be due to variations in scattering angles caused by uneven particle surfaces, fluctuations in the output power of the solid-state source, and inversion errors introduced by the network algorithm.

During the microwave drying process of low-loss particulate materials, changes in material volume can lead to variations in the equivalent permittivity, affecting the efficiency of microwave energy utilization. The algorithm predicts the height of materials, obtaining real-time information about the material’s status, which is conducive to reducing reflection and improving efficiency by monitoring the height. Additionally, because low-loss substances have a low microwave absorption efficiency, multi-port reflection power measurements can provide effective amplitude signals to characterize the microwave scattering behavior of materials with different volumes.

This system offers a cost-effective power measurement strategy that employs the residual-MLP algorithm to monitor changes in particle volume fraction in real-time and identify optimal working conditions. In the future, the measurement method of reflection power can be utilized in microwave drying as a power control sensor, which is integrated into an input channel without an external S-parameter measurement instrumentation. Upon variations in the morphology, quality, and category of dry materials, the system orchestrates real-time recalibration of the drying protocol, leveraging power metrics relayed by sensor feedback. The proposed power measurement system and network algorithms may replace traditional VNA measurements, providing an economical and convenient solution for microwave measurements and energy applications in industrial production.

## Data Availability

The original contributions presented in this study are included in the article. Further inquiries can be directed to the corresponding author.

## Abbreviations

The following abbreviations are used in this manuscript:

MLP	Multilayer Perceptron
PET	Polyethylene Terephthalate
VNA	Vector Network Analyzer
